# Food Consumed by High School Students during the School Day

**DOI:** 10.3390/nu12020485

**Published:** 2020-02-14

**Authors:** Almudena Garrido-Fernández, Francisca María García-Padilla, José Luis Sánchez-Ramos, Juan Gómez-Salgado, Gabriel H. Travé-González, Elena Sosa-Cordobés

**Affiliations:** 1 Department of Nursing, University of Huelva, 21007 Huelva, Spain; almudena.garrido@denf.uhu.es (A.G.-F.); jsanchez@denf.uhu.es (J.L.S.-R.); 2 Department of Sociology, Social Work and Public Health, University of Huelva, 21007 Huelva, Spain; 3 Safety and Health Postgraduate Programme, Universidad Espíritu Santo, 092301 Guayaquil, Ecuador; 4 Department of Pedagogy, University of Huelva, 21007 Huelva, Spain; gabriel.trave@dedu.uhu.es; 5 Doctoral Programme, University of Huelva, 21007 Huelva, Spain; elenasosacordobes@gmail.com

**Keywords:** nutrition, adolescent, breakfast, school

## Abstract

The development of healthy eating habits in adolescence is perceived as an effective strategy to avoid health problems in adulthood. The involvement of educational centres’ governing boards, as well as the Educational State and Regional Administrations’, may be necessary to create healthy food environments during the school day. The objective of this study is to identify the relationship between students’ eating habits during the school day and sociodemographic, family and physical activity variables, as well as the existence of a school cafeteria. For this, a cross-sectional study in a stratified random sample of 8068 students of Public Secondary Education High Schools of Andalusia (Spain) has been carried out. The results show that students who are 14 years old or older are more likely to skip breakfast at home (odds ratio (OR): 1.81, 95% confidence interval (CI): 1.55–2.12) than those under this age. Students whose mothers do not have a university education are more likely to consume incomplete breakfasts (OR: 1.83, 95% CI: 1.26–2.65). Snacks with sweets (OR: 1.93, 95% CI: 1.67–2.23), candy in general (OR: 2.75, 95% CI: 2.38–3.19), and bagged crisps (OR: 3.06, 95% CI: 2.65–3.54) were more likely to be consumed in schools with a cafeteria. The factors that significantly influence the eating habits of secondary students in Andalusia include age, sex, parental level of education, physical activity and the existence of a cafeteria.

## 1. Introduction

The regular practice of physical activity and adequate nutrition are fundamental habits for improving quality of life [1] and reducing some public health problems that affect the population worldwide, such as being overweight and obesity; these problems are closely related to low-quality diets and sedentary lifestyles [2,3,4,5,6]. With regard to eating, diverse studies suggest that the progressive loss of a varied and balanced traditional diet can lead to an overweight condition and obesity. In the Spanish adolescent population, the consumed food deviates from the Mediterranean diet by exceeding the caloric intake because of an excessive consumption of meat, fat and sweets and a shortage of fruits, cereals, legumes, and fish [2,3,4,5,6].

Several studies have been performed in Spain that confirm the significant impact these unhealthy habits have on the population [2,7,8]. Currently, there is also significant awareness regarding healthy eating habits [9,10,11,12]. School age is an important time to develop healthy eating and physical activity habits, which improve the feeling of well-being by more successfully developing school activities and reducing the risk of chronic diseases in adulthood [13,14]. An increasing number of studies correlate poor physical and intellectual performance with inadequate caloric intake, low nutritional quality in their diet and the omission of breakfast [7,15]. Previous studies have shown that when young people eliminate breakfast or consume an incomplete breakfast, they may compensate by consuming unhealthy products, many of which are purchased in the school cafeteria itself [16].

These findings suggest that institutions must ensure an affordable food supply of acceptable nutritional quality in the educational environment and encourage educational interventions aimed at achieving this objective. The training and involvement of different educational and community agents in the promotion of a healthy diet in secondary education high schools (SEHS) is a high priority [17].

Healthy-eating education seems to occupy an insignificant place in the secondary school curriculum. In addition, fundamentally prescriptive approaches and ineffective recommendations are used with respect to substantial changes in student habits [18]. Finally, in a more recent study in which the role of the family was analysed, parents were reported to emphasize the importance of education in the acquisition of healthy habits and in the responsibility and control on the part of the family, although they identify certain difficulties such as lack of time, convenience and the negative influence of the market [19]. These results are relevant for the present study as they show family-related variables such as type of family, parents’ level of education and employment situation, which have been used here as independent variables.

This study is integrated into the ANDALIES project and is dedicated to the promotion of healthy eating in the school environment. A summary of the research has been distributed in secondary education centres in Andalusia [20].

### Objectives

In the context of the SEHS of Andalusia (Spain), the objective of this study was to explore the relationships between students’ eating habits during the school day, sociodemographic and family characteristics, the existence of a cafeteria in the educational centre, and the practice of physical activity. The time frequency comprises the time lapse between 8 am and 3 pm.

## 2. Materials and Methods

### 2.1. Design and Sample

This was a cross-sectional exploratory study based on a multistage sample stratified by clusters. The study population was randomly selected and stratified by province and size of town of residence of the 95 SEHS. The students were chosen by the systematic sampling of classrooms. The sample comprised 8068 students, assuming a sampling error of ±2% for a confidence level of 95.5% in the estimate of the prevalence of dietary habits during the school day. After the data collection, an SEHS has been eliminated as the students’ characteristics greatly differed from the rest of the sample. This is a centre which is located in the South of Andalusia, in the province of Cadiz, where a high proportion of immigrants from Northern Africa arrive. These have dietary habits and sociodemographic characteristics that greatly differ from the rest of the Andalusian population. Thus, of the 95 initially selected centres, only 94 remained under study.

Information on the sizes of each aggregate and stage was obtained from the database of the Council of Education of the Andalusia Council and the Confederation of Parent Associations for Public Education (*Confederación de Asociaciones de Padres y Madres por la Enseñanza Pública CODAPA*).

Reserve samples were selected to replace the secondary schools that refused to participate, by the same primary selection procedure. Only 6 centres have been replaced: 3 for refusing to participate in the study, 2 due to errors in the databases (non-existent centres), and 1 due to methodological criteria (the sampling was stratified by province and, in one of the provinces, many centres were found without a school cafeteria. The methodological criteria followed was to substitute one of these centres by one from our reserve sample that had a school cafeteria).

### 2.2. Variables and Data Collection

The following information on eating habits during the school day was collected: breakfast at home and type of breakfast; intake at school and characteristics of the school snack; and the consumption of candy, bagged crisps, and pastries (sugary products and products high in salt and saturated fats, which are not recommended for a healthy diet). In addition, information was obtained on sociodemographic and family characteristics (age, sex, province, parents educational level, type of family, parents’ employment situation), the existence of a cafeteria in the school, physical activity performed, and self-assessment of the students’ physical activity levels. In case there is no school cafeteria available in the centre, students bring their own snack or buy it in nearby shops.

The questionnaire was self-administered and was previously submitted for review by expert judges and applied to a sample of 30 students from different populations, sexes, ages and social statuses. The existence of a cafeteria in the SEHS was verified by observation. To inform and request access to the school, the Provincial Delegations of Education were contacted. Subsequently, visits to the SEHS were arranged with a school official from the centre’s management team by telephone or email.

The questionnaire was composed of 13 questions, where three of them were open-ended and the remaining 10 were closed-ended, with several possible answers. These questions surveyed information on the students’ sociodemographic and family characteristics (8 questions), and on their dietary habits for breakfast and during their school day (5 questions). The questions surveyed about “generally” or “typically” during the school day and asked for a typical meal (see Supplementary Files 3.1 and 3.2). Given the variability of the types of breakfast and afternoon snack, these data were assessed through direct responses to open questions. Three researchers categorised the types of breakfast and afternoon snack independently. As for breakfast, a consensus of 8 types of breakfast was reached by assessing the nutritional composition of the different foods declared (Table 1). In descriptive terms, these 8 types of breakfast were grouped into three categories (Table 1), and in analytical terms, they were classified as dichotomous. The dichotomisation was made by considering the variables “full breakfast” (types 1 and 2) and “incomplete breakfast” (types 3–8). The reference category was full breakfast.

As for the type of afternoon snack, 8 typologies were identified according to their ingredients, grouping them into four categories (Table 2) regarding descriptive terms. In analytical terms, these were dichotomised in an afternoon snack without sweets (types 1–3), and an afternoon snack including sweets (types 4–8). The reference category was snack without sweets.

The variables sweets and processed baked goods consumption, bagged crisps consumption, and candy consumption were independently measured through a specific question in the questionnaire (question 11 on the attached questionnaire).

### 2.3. Data Analysis

The data were analysed using the statistical package SPSS version 19. The percentages of each eating habit were obtained for the descriptive analysis. For the relationships between variables, through the descriptive data in Table 3 and Table 4, a univariate logistic regression was used for each of the seven dietary habits previously dichotomised, assessing the predictive role of the sociodemographic variables, physical activity, existence of school cafeteria, and relatives.

To estimate the unbiased influence of each of the factors on eating habits, seven multiple logistic regression analysis were performed, using as predictive variables those which showed more relevance in the initial analysis and a significance level of relationship of *p* < 0.1. Odds ratio (OR) associated to each factor, with 95% confidence interval for each habit, were estimated.

### 2.4. Ethical Considerations

The approval of the Research Ethics Committee of the Public Health System of Andalusia in Huelva was obtained, as well as the approval of the Bioethics Committee of the University of Huelva. Participation in the study was voluntary and free. An informed consent form was delivered to the participants to be signed by their legal guardians, ensuring the confidentiality of the data and their exclusive use for this study. (See Supplementary Files 1.1, 1.2, 2.1 and 2.2)

## 3. Results

Of the total sample, 50.9% were boys and 49.1% were girls. The average age of the surveyed students was 15.71 years (standard deviation (SD) = 3.65). A total of 65.4% of the students attended compulsory secondary education, 20.9% were baccalaureate students, and 13.6% were in another type of educational programme. The most common type of family was a first-generation nuclear family (71.8%). In terms of physical activity, 5891 students (73%) reported to perform physical activity outside school.

The descriptive data related to the study variables can be seen in Table 3 and Table 4. It is worth highlighting that a total of 78.2% of the students had breakfast at home, although it was incomplete for 74.1% due to low caloric intake. Recess school snacks included sandwiches (90.8%), packaged juices (63.3%) and a large variety of bagged crisps, industrial pastries and sweets (75%). Table 5 shows the results of the crude analysis of the different factors associated with dietary habits during the school day. From these results obtained from the tables, the predictive variables included in the multiple regression analysis in Table 6 are identified.

### 3.1. Skipping Breakfast at Home

In the multiple regression analysis (Table 6), students aged 14 years and older had a higher probability of not having breakfast before going to school than students aged 12 and 13 years old, with an OR of 1.81 (95% CI: 1.55–2.12). Moreover, girls (OR: 1.43, 95% CI: 1.25–1.62) and students who belonged to families other than those considered first-generation or extended (OR: 1.44, 95% CI: 1.23–1.69) had the highest probability of not having breakfast. The presence of a cafeteria, although to a lesser degree, increases the probability of not having breakfast at home before going to school (OR: 1.23, 95% CI: 1.03–1.46). Those who did not perform physical activity had a higher probability of not having breakfast (OR: 1.46, 95% CI: 1.27–1.68).

### 3.2. Incomplete Breakfast

The probability of consuming an incomplete breakfast was 41% lower in students aged 14 and older (OR: 0.59, 95% CI: 0.40–0.85). The students whose mothers did not have a university education had a higher probability of having an incomplete breakfast (OR: 1.83, 95% CI: 1.26–2.65), as did students whose both parents did not have a university education (OR: 1.48, 95% CI: 1.01–2.17). There was a greater probability of consuming an incomplete breakfast if the student did not perform physical activity (OR: 1.74, 95% CI: 1.09–2.76) (Table 6).

### 3.3. No Snacks at School

The probability of not having a snack in school was somewhat higher for students aged 14 or older (OR: 1.19, 95% CI: 1.05–1.36) and whose families were other than first-generation or extended (OR: 1. 32, 95% CI: 1.15–1.52). The presence of a cafeteria also increased this probability (OR: 1.28, 95% CI: 1.10–1.50). There was a weaker association with parents’ employment situations. The students whose parents were unemployed had a higher probability of not snacking at school (OR: 1.16, 95% CI: 1.01–1.34) (Table 6).

### 3.4. Type of Snack

The existence of a cafeteria increased the probability of consumption of unhealthy school meals (OR: 1.93, 95% CI: 1.67–2.23). Students aged 14 and older had a somewhat higher probability of consuming this type of snack (OR: 1.16, 95% CI: 1.04–1.30), as did students who did not perform physical activity outside the school (OR: 1.14, 95% CI: 1.01–1.28). Belonging to the provinces of eastern Andalusia decreased this probability by 15% (OR: 0.85, 95% CI: 0.77–0.93) (Table 6).

### 3.5. Consumption of Bagged Crisps

The existence of a cafeteria in the school was strongly related to the consumption of bagged crisps, with an OR of 3.06 (95% CI: 2.65–3.54). Girls were more likely to consume bagged crisps during the school day (OR: 1.36, 95% CI: 1.23–1.50) (Table 6).

### 3.6. Consumption of Candy

The existence of a cafeteria was once again related to a greater likelihood of consuming candy at school (OR: 2.75, 95% CI: 2.38–3.19). Students aged 14 years and older had an 18% lower probability of consuming candy than those under 14 years of age (OR: 0.82, 95% CI: 0.73–0.93). This probability was also 35% lower in those who belonged to the provinces of eastern Andalusia (OR: 0.65, 95% CI: 0.58–0.71). Meanwhile, girls were more likely to consume candy than boys (OR: 1.48; 95% CI: 1.33–1.64), as were students whose mothers and fathers did not have a university education (OR: 1.25, 95% CI: 1.08–1.45 and OR: 1.20, 95% CI: 1.02–1.38, respectively) (Table 6).

### 3.7. Consumption of Sweets and Pastries

Students belonging to the provinces of eastern Andalusia had a higher probability of consuming sweets and pastries (OR: 1.56, 95% CI: 1.41–1.73), as did students belonging to families other than first-generation or extended (OR: 1.38, 95% CI: 1.20–1.58). In schools with a cafeteria, there was a 20% lower probability of consuming sweets and pastries than in schools that lacked a cafeteria (OR: 0.80; 95% CI: 0.68–0.90). There was a weaker relationship between the level of education and the employment situation of the parents. Students whose parents did not have a university education (OR: 1.16, 95% CI: 1.01–1.33) and students whose parents were unemployed (OR: 1.15, 95% CI: 1.00–1.32) were more likely to consume sweets and pastries (Table 6).

## 4. Discussion

The data in this study show that age influenced the different studied eating habits, particularly regarding eating breakfast at home and consuming candy. This factor was also associated with the type of breakfast the students consumed. Students over 14 years were more likely to skip breakfast and not snack in the SEHS, although students younger than 14 consumed incomplete breakfasts and candy the most. Performing regular physical activity outside the school resulted in the acquisition of other healthy habits, such as healthy eating habits. Thus, students who performed physical activity ate breakfast at home more frequently than those who did not and were more likely to consume a complete breakfast.

The existence of a cafeteria in the school had a strong association with the type of snack that students consumed, particularly regarding the intake of candy and bagged crisps. Likewise, there was an inverse association between the existence of a cafeteria and the consumption of sweets and pastries, with schools having a cafeteria seeing a lower consumption of sweets and pastries. This observation can be attributed to greater sensitivity to recommendations by public administrations, media and/or schools to reduce the consumption of industrial baked goods. These entities have decreased the supply of such products in school cafeterias to lower consumption [21] but have not been as sensitive regarding other non-recommended products (candy or bagged crisps). Therefore, we urge that the recommendations be followed and that the provisions of the Spanish Food Safety and Nutrition Act be met to reduce consumption of the remaining non-recommended products. In one of the reviewed articles, where selling healthy products at school is proposed, we find satisfaction among the students, creating a positive effect on healthy food accessibility [22].

Finally, we emphasise that the educational level of the mother and father is closely related to the type of breakfast consumed before going to school, as well as the consumption of candy. Students whose mothers and fathers did not have a university education were more likely to have incomplete breakfasts at home and to consume sweets at school.

The data obtained in this study are self-declared, so they share the usual limitation of data quality regarding self-declared food intake, in this case. A possible weakness of this study is the lack of questionnaire responses regarding the parents’ professions due to ignorance or confusion about their real professions. Additionally, the participants reported the contents of a “typical” breakfast, and so within-person variability in food consumption was not examined. Certain discrepancies between the answers to “Snack in SEHS” and “Type of snack” are possible, as they were obtained from two different questions of the questionnaire. Because this is a cross-sectional study, the relationships found cannot be considered causal. Among the strengths of the study we can highlight the sample size, as well as its representative capacity for the whole Andalusian region. As far as we are concerned, this is the first study specifically aimed at knowing the dietary habits of secondary education students during the school day.

Most of the studies reviewed were based on the evaluation of breakfast [13,23,24,25,26,27,28,29,30], on the analysis of adolescents’ daily consumption or on overall eating habits [31,32,33,34,35,36,37,38] and on the relationship of diet with physical activity [39,40,41,42] or with school performance [15,43,44,45]. We also found some educational interventions [42,46,47,48,49,50,51,52] and studies that focused on adherence to the Mediterranean diet [53,54]. Of special interest are those relating emotional state [55] or social support [56,57] with breakfast among adolescents. The scarcity of research on adolescent eating habits during the school day [16,17,18,19,20,56] justifies the relevance of this line of research and supports the need for policies that favour the creation of healthy environments.

As mentioned above, we have not identified previous studies that focus on the eating habits of young people during the school day. Therefore, we consider this study to provide novel data that can be a reference for promoting healthy eating habits in the school environment.

Our results agree with those of other studies that high school students eat some breakfast at home [27,29,39,40] and that a large portion of them eat a poor breakfast [15,30,33,39]. Moreover, as in our study, age and sex are predictive factors of omitting breakfast or having breakfast at home before going to the SEHS. Several studies show that girls tend to skip breakfast more frequently than boys, as well as older students as compared to younger ones [15,23,29]. This last fact is also widespread among those students whose parents have a higher educational level [55] and among those who do not perform physical activity [55].

Most reviewed studies conclude that having a full and healthy breakfast is vital for the mental and physical health status of secondary education students. Also, performing interventions aimed at these practices are both necessary and effective, although there is little research on the importance of school snacks, the consumption of non-recommended products during the school day, or the real need for school cafeterias, thus stating breakfast as the key and only responsible element composing dietary health regarding the school day.

Knowing about dietary habits during adolescence, related social determinants, regulations and resources of the school will be very useful in the design of plans and strategies towards health promotion. Performing interventions that consolidate good lifestyle habits, that create health-promoting environments and decrease the risks associated with unhealthy eating in adulthood is a commitment for the future in the context of community health. Moreover, the creation and continuity of future research must continue to focus on adolescent eating habits during the school day and take into account the other agents involved, including family, teachers, administration, parents’ associations and community health teams.

## 5. Conclusions

The factors that significantly influence the eating habits of secondary students in Andalusia include age, sex, parental level of education, physical activity and the existence of a cafeteria.

More concretely, among the dietary habits of students during the school day, the following associations can be highlighted:

Skips breakfast at home: students over 14 years old, girls, students who do not perform physical activity outside the school and those who belong to a family other than first-generation or extended tend to more frequently skip breakfast at home before going to school. In schools with a cafeteria, students eat less at home.

Consumes incomplete breakfast: students under 14 years of age who do not perform physical activity or whose parents do not have a university education consume an incomplete breakfast more frequently.

Does not snack at the SEHS: there is a greater probability of not snacking at school for students aged 14 or older, those with an unemployed father and those who belong to families other than the extended or first-generation families. Curiously, there is less snacking in schools with a cafeteria than in schools without a cafeteria.

Type of snack: unhealthy snacks are consumed more frequently among students whose schools have a cafeteria and among those in western Andalusia. Students who are 14 years or older and those who do not perform physical activity have the highest probability of consuming unhealthy snacks.

Consumption of bagged crisps: the consumption of bagged crisps is higher in schools with a school cafeteria and in girls.

Consumption of candy: having a cafeteria in the SEHS also increases the consumption of candy. The study sample presents a greater consumption of candy in girls, students under 14 years of age, those whose parents do not have a university education and those from western Andalusia.

Consumption of sweets and pastries: the probability of consuming sweets and pastries during the school day is greater in the provinces of eastern Andalusia, in students belonging to families other than first-generation or extended families and in students whose fathers do not have a university education or are unemployed. Consumption is lower in schools with a cafeteria.

## Figures and Tables

**Table 1:** Type of breakfast.

Category	Type	Designation	Ingredients
Full breakfast (B1)	1	Full breakfast with bread or breakfast cereals	Milk drink, bread (toast or sandwich) and a piece of fruit or natural fruit juice
	2	Full breakfast with pastries	Milk drink, biscuits or breakfast cereals and a piece of fruit or natural fruit juice
Incomplete breakfast (B2)	3	Incomplete breakfast with bread and/or breakfast cereals	Drink (milk or juice…) and a toast or sandwich
	4	Incomplete breakfast with pastries	Drink and any pastry (muffins, sponge cake or biscuits)
	5	Incomplete breakfast with pastries or bread	Drink (milk or juice…), bread and/or breakfast cereals and pastries (including processed baked goods)
	6	Solid incomplete breakfast	Solid food, may include a toast, sandwich or piece of fruit
	7	Liquid incomplete breakfast	Liquid food, may be milk products like milk, milk with cocoa powder, liquid yogurt, or juice
Low nutritional quality breakfast (B3)	8	Low nutritional quality breakfast	Fizzy drink and/or crisps and/or processed baked goods, or any other product which is not recommended in a healthy diet

**Table 2:** Type of afternoon snack.

Category	Type	Designation	Ingredients
Afternoon snack without sweets (S1)	1	Solid and liquid food	A combination of high nutritional quality food and food which could be improved, plus a liquid product
	2	Solid and liquid food, plus fruit	A combination of the previous group, plus a piece of fruit
	3	Food from three groups	A combination of food from three groups: cereals + fruit + diary product
Afternoon snack including sweets (S2)	4	Solid food plus non-recommended products	A combination of solid food (sandwich) and processed baked goods, pastries, crisps and/or candy
	5	Solid and liquid food plus non-recommended products	A combination of the previous group plus a liquid product
	6	Liquid food plus non-recommended products	A combination of liquid food plus processed baked goods, pastries, crisps and/or candy
Liquid varied afternoon snack (S3)	7	Liquid food	Only liquid food: milk drink, juice…
Only sweets (S4)	8	Only non-healthy food	Only candy, crisps, both, or processed baked goods

**Table 3:** Eating habits according to sociodemographic characteristics, the existence of a cafeteria and physical activity.

		Breakfast at Home n %	Type of Breakfast (B1, B2, B3) n %			Snack in the SEHS n %	Type of Snack (S1, S2, S3, S4) n %				Candy Consumption n %	Bagged Crisps Consumption n %	Sweets and Pastries Consumption n %
Age	12–13	1743	42	1654	7	1575	1198	559	77	21	1094	963	614
		86.2	2.5	97.1	0.4	78.2	64.6	30.1	4.2	1.1	56.3	50.6	32.4
	14–16	2775	88	2583	9	2679	1943	1036	161	49	1912	1677	1223
		78	3.3	96.4	0.3	75.3	60.9	32.5	5	1.5	56.2	50.1	36.2
	>16	1766	80	1604	6	1792	1294	662	184	30	1048	1117	902
		71.8	4.7	94.9	0.4	72.8	59.6	30.5	8.5	1.4	44.7	47.7	38.3
Sex	Male	3340	132	3072	15	3094	2334	1146	202	48	1875	1775	1340
		82	4.1	95.4	0.5	76.1	62.6	30.7	5.4	1.3	48.3	45.9	34.6
	Female	2915	77	2742	7	2920	2081	1099	219	52	2166	1971	1391
		74.2	2.7	97	0.2	74.3	60.3	31.8	6.3	1.5	57.3	53.3	37.3
Province	Huelva	783	19	736	3	638	383	282	92	44	496	456	168
		83.7	2.5	97.1	0.4	68.3	47.8	35.2	11.55	5.5	56.7	52.6	19.8
	Almería	700	30	624	0	632	444	213	90	11	232	251	239
		79.9	4.6	95.4	0	72.4	58.6	28.1	11.9	1.5	27.9	30.8	28.3
	Cádiz	733	26	672	3	774	606	269	37	9	597	553	271
		74.9	3.7	95.9	0.4	79	65.8	29.2	4	1	62.6	58.2	28.5
	Córdoba	569	17	546	0	568	434	235	21	7	376	361	199
		78.1	3	97	0	78.1	62.3	33.7	3	1	53.2	52	28.5
	Sevilla	1373	39	1284	11	1353	996	520	59	15	917	974	652
		77.2	2.9	96.3	0.8	76.2	62.6	32.7	3.7	0.9	54.9	58.6	39.5
	Málaga	970	35	906	3	1012	904	291	37	2	649	589	538
		75.1	3.7	96	0.3	78.1	73.3	23.6	3	0.2	52.2	47.8	43.2
	Granada	659	26	607	2	615	414	233	43	4	408	344	385
		79.7	4.1	95.6	0.3	74.3	59.7	33.6	6.2	0.6	51.2	44.3	48.7
	Jaén	497	18	466	0	454	254	214	43	8	379	229	287
		79.9	3.7	96.3	0	72.8	48.9	41.2	8.3	1.5	62.2	38.4	47.6
Cafeteria	Yes	5254	169	4887	18	5039	3604	2007	374	97	3647	3437	2279
		77.6	3.3	96.3	0.4	74.5	59.3	33	6.1	1.6	56	53.4	35.3
	No	1030	41	654	4	1007	831	250	48	3	407	320	460
		81	4.1	95.5	0.4	79.3	73.4	22.1	4.2	0.3	34.7	27.7	39.1
Outside physical activity	Yes	4765	180	4401	19	4462	3328	1634	290	69	2943	2735	1974
		80.8	3.9	95.7	0.45	75.7	62.5	30.7	5.5	1.3	51.9	48.8	35.1
	No	1418	28	1347	3	1495	1047	587	124	29	1061	973	724
		70.3	2	97.8	0.2	74	58.6	32.8	6.9	1.6	55	51.4	37.7
Physical activity self-evaluation	Sedentary	713	16	670	1	743	519	269	63	12	464	471	348
		72.5	2.3	97.5	0.1	75.8	60.1	31.2	7.3	1.4	49.6	50.6	36.9
	Moderate	1681	74	1547	6	1614	1178	587	123	26	1068	1015	733
		78.6	4.5	95.1	0.4	75.4	61.5	30.7	6.4	1.4	52	49.7	35.7
	Active	1771	54	1661	4	1681	1248	641	94	22	1108	1010	739
		79.6	3.1	96.6	0.2	75.6	62.2	32	4.7	1.1	51.8	48.2	35
	Very active	1109	51	1013	8	1048	799	371	64	22	697	643	471
		81.2	4.8	94.5	0.7	76.8	63.6	29.5	5.1	1.8	53.6	50.3	36.7
	Don’t know	837	11	787	3	797	572	329	64	17	619	530	374
		75.9	1.4	96.3	0.4	72	58.2	33.5	6.5	1.7	58.5	50.6	35.9

B1: Complete breakfast; B2: Incomplete breakfast; B3: Breakfast of low nutritional quality. S1: Combined snack without sweets (healthy snack); S2: Combined snack with sweets; S3: Varied liquid snack; S4: Exclusive ingestion of sweets. SEHS: Secondary Education High Schools. Missing values: Breakfast at home (31) Type of breakfast (1810) Snack in the SEHS (31) Type of snack (854) Candy consumption (378) Bagged crisps consumption (471) Sweets and pastries consumption (437).

**Table 4:** Eating habits according to family characteristics.

		Breakfast at Home n %	Type of Breakfast (B1, B2, B3) n %			Snack in the SEHS n %	Type of Snack (S1, S2, S3, S4) n %				Candy Consumption n %	Bagged Crisps Consumption n %	Sweets and Pastries Consumption n %
Mother’s education	Does not know how to read/write	39	2	35	0	34	18	16	1	1	33	33	27
		72.2	5.4	96.4	0	63	50	44.4	2.8	2.8	62.3	63.5	51.9
	No education	259	4	247	2	257	198	104	19	6	199	172	134
		73.8	1.6	97.6	0.8	72	60.6	31.8	5.8	1.8	59.2	52.1	40.6
	Incomplete primary education	547	12	515	1	534	412	199	39	12	397	342	248
		74.4	2.3	97.5	0.2	72.8	62.2	30.1	5.9	1.8	56.5	49.1	35.4
	Primary education	1568	36	1482	3	1530	1140	544	101	31	1094	973	672
		77.3	2.4	97.4	0.2	75.5	62.8	30	5.6	1.7	56.2	50.5	34.9
	Secondary education	2429	79	2259	14	2365	1704	879	168	34	1556	1465	1071
		78.6	3.4	96	0.6	76.8	61.2	31.6	6	1.2	52.7	50.4	36.6
	University education	1106	68	994	1	1010	713	409	56	12	590	597	437
		82.5	6.4	93.5	0.1	75.4	59.9	34.4	4.7	1	45.9	46.7	34.1
Father’s education	Does not know how to read/write	22	0	20	0	25	16	12	2	0	24	18	16
		61.1	0	100	0	69.4	53.3	40	6.7	0	64.9	52.9	44.4
	No education	268	6	253	2	268	198	111	20	6	206	172	144
		73.2	2.3	96.9	0.8	72.2	59.1	33.1	6	1.8	59.2	49.6	42
	Incomplete primary education	640	17	601	1	635	463	227	46	16	463	399	291
		76.4	2.7	97.1	0.2	75.7	61.6	30.2	6.1	2.1	57.7	50	36.2
	Primary education	1483	38	1398	3	1474	1061	533	102	34	1066	943	672
		76.7	2.6	97.2	0.2	76.2	61.3	30.8	5.9	2	57.2	51.3	36.4
	Secondary education	2284	71	2138	10	2181	1622	811	144	29	1416	1355	961
		79.4	3.2	96.3	0.5	75.9	62.2	31.1	5.5	1.1	51.7	50.2	35.3
	University education	1043	61	934	2	960	674	372	56	8	564	579	391
		83	6.1	93.7	0.2	76.6	61.5	33.5	5	0.7	46.6	48.2	32.8
Family type	1st gen. nuclear fam	4602	148	4315	14	4403	3213	1645	280	73	2952	2691	1846
		80	3.3	96.4	0.3	76.6	61.7	31.6	5.4	1.4	53.4	49.4	33.8
	Extended family	397	12	369	1	386	269	137	26	4	249	226	193
		78.3	3.1	96.6	0.3	76.1	61.7	31.4	6	0.9	53	47.9	40.3
	Single parent	532	21	479	5	506	384	204	40	7	355	351	310
		72.5	4.2	94.9	1	68.9	60.5	32.1	6.3	1.1	50.4	50.75	44.9
	2nd gen. nuclear family	211	3	196	1	187	148	85	15	4	142	142	117
		72.8	1.5	98	0.5	64.7	58.7	33.7	6	1.6	51.8	53	43
	Only grandparents	151	4	140	0	161	116	61	5	6	120	108	61
		75.5	2.8	97.2	0	80.5	61.7	32.4	2.7	3.2	62.5	56.2	32.3
	Other	375	21	327	1	382	296	115	53	5	226	232	211
		70.4	6	93.7	0.3	71.8	63.1	24.5	11.3	1.1	44.8	46.2	41.3
Mother’s employment	Working	3336	113	3110	12	3187	2301	1203	211	55	2109	1986	1448
		79.2	3.5	96.1	0.4	75.8	61	31.9	5.6	1.5	52.5	50	36.3
	Unemployed	630	25	583	4	601	469	234	43	6	446	383	310
		74.9	4.1	95.3	0.7	71.5	62.4	31.1	5.7	0.8	55.4	48.3	38.7
	Domestic chores	2114	62	1973	6	2047	1503	752	137	38	1390	1260	862
		78.6	3	96.7	0.3	76.1	61.9	30.9	5.6	1.6	53.6	49.4	33.7
	Retired	24	1	22	0	24	21	7	3	0	12	14	12
		72.7	4.3	95.7	0	72.7	67.7	22.6	9.7	0	36.4	42.4	36.4
	Student	4	1	3	0	5	3	1	0	0	4	2	2
		80	25	75	0	100	75	25	0	0	80	40	40
Father’s employment	Working	4760	153	4461	16	4572	3303	1717	300	85	3032	2843	1974
		79.5	3.3	96.3	0.3	76.3	61.1	31.8	5.6	1.6	52.8	50	34.7
	Unemployed	898	31	826	2	868	669	316	63	7	643	562	440
		75.3	3.6	96.2	0.2	73	63.4	30	6	0.7	56.9	50.6	39
	Domestic chores	79	2	70	0	77	48	34	6	2	54	47	37
		76.7	2.8	97.2	0	74	53.3	37.8	6.7	2.2	55.1	49.5	38.1
	Retired	119	5	110	0	122	92	37	7	0	72	63	55
		77.3	4.3	95.7	0	79.7	67.6	27.2	5.1	0	48.3	43.8	37.2
	Student	2	0	2	0	2	0	2	0	0	1	1	1
		100	0	100	0	100	0	100	0	0	50	50	50

B1: Complete breakfast; B2: Incomplete breakfast; B3: Breakfast of low nutritional quality. S1: Combined snack without sweets (healthy snack); SEHS: Secondary Education High Schools; S2: Combined snack with sweets; S3: Varied liquid snack; S4: Exclusive ingestion of sweets. Missing values: Breakfast at home (31) Type of breakfast (1810) Snack in the SEHS (31) Type of snack (854) Candy consumption (378) Bagged crisps consumption (471) Sweets and pastries consumption (437).

**Table 5:** Factors associated with eating habits during the school day (crude).

	No Breakfast at Home OR 95% CI (OR)	Incomplete Breakfast OR 95% CI (OR)	No Snack at SEHS OR 95% CI (OR)	Unhealthy Snack OR 95% CI (OR)	Candy Consumption OR 95% CI (OR)	Bagged Crisps Consumption OR 95% CI (OR)	Sweets and Pastries Consumption OR 95% CI (OR)
Age (>14 y)	**2.031** **1.76–2.33**	**0.633** **0.44–0.89**	1.170 1.05–1.29	**1.150** **1.04–1.26**	**0.823** **0.74–0.91**	0.942 0.85–1.04	**1.228** **1.10–1.37**
Sex (Girl)	**1.585** **1.42–1.76**	**1.530** **1.15–2.03**	1.102 0.96–1.22	1.10 1.00–1.21	**1.435** **1.31–1.57**	**1.342** **1.22–1.46**	1.123 1.02–1.23
Province (Eastern)	1.004 0.90–1.11	**0.745** **0.56–0.98**	1.028 0.92–1.13	0.897 0.81–0.98	**0.702** **0.64–0.76**	**0.549** **0.50–0.60**	**1.580** **1.43–1.73**
Father no university degree	**1.411** **1.20–1.65**	**2.182** **1.59–2.98**	1.052 0.91–1.21	0.963 0.84–1.09	**1.391** **1.22–1.57**	1.095 0.96–1.24	**1.166** **1.02–1.33**
Mother no university degree	**1.381** **1.18–1.61**	**2.334** **1.72–3.15**	0.994 0.86–1.14	0.927 0.81–1.05	**1.425** **1.26–1.60**	**1.165** **1.03–1.31**	1.097 0.96–1.24
Other than 1st gen and extended family	**1.527** **1.35–1.72**	0.796 0.57–1.10	**1.370** **1.21–1.54**	1.022 0.91–1.14	**1.131** **1.01–1.26**	1.045 0.93–1.16	**1.392** **1.24–1.55**
Mother unemployed	1.095 0.98–1.22	1.054 0.79–1.39	1.040 0.93–1.15	0.958 0.87–1.05	0.962 0.87–1.05	1.057 0.96–1.15	0.203 0.85–1.03
Father unemployed	**1.273** **1.01–1.47**	0.914 0.61–1.35	**1.192** **1.03–1.37**	0.907 0.79–1.03	**1.179** **1.03–1.34**	1.025 0.90–1.16	**1.207** **1.05–1.37**
SEHS cafeteria existence	**1.232** **1.05–1.43**	1.243 0.87–1.76	**1.313** **1.13–1.52**	**1.898** **1.64–2.18**	**2.396** **2.10–2.72**	**2.982** **2.59–3.42**	**0.848** **0.74–0.96**
No physical activity outside SEHS	**1.772** **1.57–1.98**	**1.968** **1.31–2.94**	1.097 0.97–1.23	**1.180** **1.05–1.31**	1.134 1.02–1.25	**1.113** **1.00–1.23**	1.115 1.00–1.24
Sedentary	**1.431** **1.23–1.66**	1.589 0.94–2.66	0.968 0.82–1.13	1.066 0.92–1.23	**0.862** **0.75–0.98**	1.047 0.91–1.20	1.054 0.91–1.21

SEHS: Secondary Education High Schools; CI = Confidence Interval; OR = Odds ratio. Bolden values = significance *p* < 0.005.

**Table 6:** Multivariate regression analysis. Factors associated with unhealthy eating habits during the school day.

	B	p	OR	Lower limit	Upper limit
				CI (OR)	CI (OR)
NO BREAKFAST AT HOME					
14 and over	0.596	**<0.001**	**1.816**	**1.552**	**2.124**
Girl	0.358	**<0.001**	**1.43**	**1.259**	**1.625**
Mother no university degree	0.17	0.078	1.185	0.981	1.431
Father no university degree	0.126	0.194	1.134	0.938	1.371
Family other than 1st gen. or extended	0.369	**<0.001**	**1.446**	**1.236**	**1.691**
Unemployed father	0.131	0.105	1.14	0.973	1.335
Cafeteria existence	0.207	**0.02**	**1.23**	**1.033**	**1.466**
No physical activity	0.384	**<0.001**	**1.468**	**1.276**	**1.688**
Sedentary	0.139	0.121	1.149	0.964	1.369
INCOMPLETE BREAKFAST					
14 and over	−0.53	**0.005**	**0.589**	**0.407**	**0.851**
Girl	0.225	0.165	1.252	0.912	1.72
Western Andalusia	−0.187	0.219	0.83	0.616	1.117
Mother no university studies	0.604	**<0.001**	**1.83**	**1.263**	**2.651**
Father no university studies	0.396	**0.042**	**1.485**	**1.015**	**2.174**
No physical activity	0.555	**0.018**	**1.741**	**1.098**	**2.761**
Sedentary	0.574	0.061	1.775	0.974	3.233
NO SNACK IN SEHS					
14 and over	0.181	**0.005**	**1.198**	**1.056**	**1.36**
Girl	0.057	0.304	1.059	0.95	1.18
Family other than 1st gen. or extended	0.283	**<0.001**	**1.327**	**1.153**	**1.528**
Father unemployed	0.156	**0.032**	**1.169**	**1.014**	**1.347**
Cafeteria existence	0.25	**0.002**	**1.284**	**1.099**	**1.499**
SNACK WITH SWEETS					
14 and over	0.152	**0.009**	**1.165**	**1.04**	**1.304**
Girl	0.06	0.247	1.062	0.959	1.176
Eastern Andalusia	−0.164	**<0.001**	**0.849**	**0.77**	**0.937**
Cafeteria existence	0.659	**<0.001**	**1.934**	**1.674**	**2.234**
No physical activity	0.136	**0.022**	**1.146**	**1.019**	**1.288**
CANDY CONSUMPTION					
14 and over	−0.189	**0.002**	**0.828**	**0.737**	**0.931**
Girl	0.395	**<0.001**	**1.485**	**1.338**	**1.648**
Eastern Andalusia	−0.432	**<0.001**	**0.649**	**0.587**	**0.719**
Mother no university degree	0.228	**0.002**	**1.256**	**1.084**	**1.456**
Father no university degree	0.177	**0.019**	**1.194**	**1.029**	**1.384**
Family other than 1st gen. or extended	0.05	0.465	1.051	0.92	1.201
Cafeteria existence	1.015	**<0.001**	**2.759**	**2.386**	**3.19**
No physical activity	0.041	0.509	1.042	0.922	1.179
Sedentary	−0.128	0.104	0.88	0.755	1.027
BAGGED CRISPS CONSUMPTION					
Girl	0.311	**<0.001**	**1.365**	**1.235**	**1.508**
Eastern Andalusia	−0.009	0.417	0.991	0.969	1.013
Mother no university degree	0.069	0.282	1.071	0.945	1.214
Cafeteria existence	1.12	**<0.001**	**3.065**	**2.653**	**3.54**
No physical activity	0.022	0.706	1.023	0.911	1.148
CONSUMPTION OF SWEETS AND PASTRIES					
14 and over	0.115	0.062	1.122	0.994	1.267
Girl	0.081	0.144	1.084	0.973	1.209
Eastern Andalusia	0.448	**<0.001**	**1.565**	**1.41**	**1.738**
Father no university degree	0.152	**0.035**	**1.164**	**1.011**	**1.339**
Family other than 1st gen. or extended	0.324	**<0.001**	**1.383**	**1.206**	**1.585**
Unemployed father	0.14	**0.046**	**1.151**	**1.003**	**1.321**
Cafeteria existence	−0.243	**<0.001**	**0.784**	**0.68**	**0.903**
No physical activity	0.076	0.238	1.079	0.951	1.223

Family other than 1st gen. or extended: Family other than first-generation or extended; SEHS: Secondary Education High Schools. The coefficients and OR of each factor are adjusted by the covariables present in each model. CI = Confidence Interval; OR = Odds ratio; p = significance level; B = regression coefficient. Bolden values = significance *p* < 0.005.

## Supplementary Materials

The following are available online at https://www.mdpi.com/2072-6643/12/2/485/s1: Supplementary File 1.1. Ethics Committee Report Spanish; Supplementary File 1.2. Ethics Committee Report English; Supplementary File 2.1 University Ethics Committee Report Spanish; Supplementary File 2.2. University Ethics Committee Report English; Supplementary File 3.1. Data collection questionnaire in Spanish; Supplementary File 3.2. Data collection questionnaire in English.
