# Supplementary material for: Food Consumed by High School Students during the School Day
Source: Nutrients. 2020 Feb 14;12(2):485. doi: 10.3390/nu12020485 (PMC7071212; doi:10.3390/nu12020485)
Supplement: Supplementary file 1 [file nutrients-12-00485-s001.zip › Supp File 3.1. Data collection questionnaire in Spanish.pdf]

“LA ALIMENTACIÓN DEL ALUMNADO DE SECUNDARIA DURANTE LA JORNADA ESCOLAR EN ANDALUCÍA”

Estimado/a estudiante:

Estamos realizando un estudio de investigación sobre la alimentación de los/as estudiantes de Secundaria, Bachillerato y Ciclos Formativos durante el tiempo que pasáis en el instituto. Necesitamos tu colaboración para poder realizar este trabajo. Para ello te pedimos, por favor, que rellenes este cuestionario de la forma más sincera posible. Por supuesto, está garantizado que tus datos personales no serán difundidos, asegurándote el total anonimato.

Marca con una CRUZ en la casilla que corresponda. En las preguntas en las que tengas que escribir, por favor, hazlo en MAYÚSCULAS para facilitar su comprensión.

Si necesitas alguna ayuda para cumplimentar el cuestionario, pídela a las personas que te lo han entregado, o al profesor/a que esté en este momento en el aula.

MUCHAS GRACIAS por tu colaboración.

codapa
CONFEDERACIÓN ANDALUZA
DE ASOCIACIONES DE MADRES Y PADRES
DEL ALUMNADO POR LA EDUCACIÓN PÚBLICA

asanec
Asociación Andaluza de Enfermería Comunitaria

1. Edad ___________años
2. Sexo: Hombre Mujer
3. Curso Académico:

 1º de Secundaria
 2º de Secundaria
 3º de Secundaria
 4º de Secundaria
 1º de Bachillerato
 2º de Bachillerato
 1º de Ciclo Formativo Grado Medio
 2º de Ciclo Formativo Grado Medio
 1º de Ciclo Formativo Grado Superior
 2º de Ciclo Formativo Grado Superior

4. ¿Con quién vives? (Marca todas las opciones posibles)

 Padre
 Madre
 Hermano/a
 Abuelo
 Abuela
 Padrastro/madrastra
 Primo/a
 Otros/as (Especificar)______________________

5. Indica la situación laboral de los adultos responsables de ti:

Padre/Tutor

 Está trabajando
 Está en paro
 Sólo se dedica a las tareas de la casa

Madre/Tutora

 Está trabajando
 Está en paro
 Sólo se dedica a las tareas de la casa

6. Indica los estudios que han realizado los adultos responsables de ti.

Padre/Tutor

 No sabe leer o escribir
 Sin estudios
 Estudios Primarios incompletos
 Estudios Primarios completos
 Estudios Secundarios
 Estudios Universitarios

Madre/Tutora

 No sabe leer o escribir
 Sin estudios
 Estudios Primarios incompletos
 Estudios Primarios completos
 Estudios Secundarios
 Estudios Universitarios

7. ¿Desayunas en casa antes de ir al Instituto? ☐ SI ☐ NO

8. Si has contestado que SÍ en la pregunta anterior ¿Qué desayunas?
____
____
____

9. ¿Comes y/o bebes algo en el Instituto durante la mañana?

☐ Siempre ☐ A veces ☐ Nunca

10. ¿Qué sueles comer y/o beber?
Indícalo____

11. Durante la mañana en el Instituto ¿Consumes alguno de estos productos?

Dulces y/o Bollería ☐ Nunca ☐ Siempre ☐ A veces ¿Cuál? ______________________________

Paquetes/Snacks ☐ Nunca ☐ Siempre ☐ A veces Específica de qué es ____ ______________

Chucherías ☐ Nunca ☐ Siempre ☐ A veces ¿Cuál? ____________________________________

12. ¿Realizas Ejercicio Físico **fuera del horario del Instituto**?

☐ Sí ☐ NO

13. ¿Cómo describirías tu actividad física diaria?

☐ Sedentaria ☐ Moderada ☐ Activa

☐ Muy Activa ☐ No sé

MUCHAS GRACIAS
