# Supplementary material for: Food Consumed by High School Students during the School Day
Source: Nutrients. 2020 Feb 14;12(2):485. doi: 10.3390/nu12020485 (PMC7071212; doi:10.3390/nu12020485)
Supplement: Supplementary file 1 [file nutrients-12-00485-s001.zip › Supp File 3.2. Data collection questionnaire in English.pdf]

"FOOD CONSUMED BY HIGH SCHOOL STUDENTS DURING THE SCHOOL DAY IN ANDALUSIA"

Dear Student:

We are conducting a research study on the dietary habits of Secondary Education, Higher Secondary Education, and Vocational Training students during the school day. We need your collaboration to be able to carry out this work. To do this, please fill out this questionnaire as honestly as possible. Of course, it is guaranteed that your personal data will not be disseminated, ensuring total anonymity.

Mark with a CROSS the appropriate box. For questions you need to write on, please do so in CAPITAL LETTERS so as to make them easier to understand.

If you need any help with completing the questionnaire, ask the people who have given it to you, or the teacher who is currently in the classroom.

THAK YOU VERY MUCH for your collaboration

[sign: CODAPA/Andalusian confederation of associations of mothers and fathers of students for public education]

[sign: asanec/Community Nursing Andalusian Association]

1. Age________________years old
2. Sex: ☐ Male ☐ Female
3. Academic course:

☐ 1st Secondary Education
☐ 2nd Secondary Education
☐ 3rd Secondary Education
☐ 4th Secondary Education
☐ 1st Higher Sec. Educ.
☐ 2nd Higher Sec. Educ.
☐ 1st Medium-level Vocational Training
☐ 2nd Medium-level Vocational Training
☐ 1st Higher-level Vocational Training
☐ 2nd Higher-level Vocational Training

4. Who do you live with? (tick all the corresponding options)

☐ Father
☐ Brother/sister
☐ Grandmother
☐ Cousin
☐ Other (specify)_______________
☐ Mother
☐ Grandfather
☐ Stepfather/Stepmother

5. Specify the employment situation of the persons responsible for you.

Father/Tutor

☐ Working
☐ Unemployed
☐ Domestic chores

Mother/Tutor

☐ Working
☐ Unemployed
☐ Domestic chores

6. Specify the educational level of the persons responsible for you

Father/Tutor

☐ Does not read/write
☐ No studies
☐ Incomplete Primary Studies
☐ Complete Primary Studies
☐ Secondary Studies
☐ University Studies

Mother/Tutor

☐ Does not read/write
☐ No studies
☐ Incomplete Primary Studies
☐ Complete Primary Studies
☐ Secondary Studies
☐ University Studies

7. Do you have breakfast at home before leaving for school? ☐ YES ☐ NO
8. If your answer was YES, what do you have for breakfast?

____
____
____

9. Do you eat and/or drink anything at school during the morning and afternoon?
☐ Always ☐ Sometimes ☐Never

10. What do you usually eat/drink?

Specify____

11. During the school day, do you consume any of these products?

Pastries/processed baked goods ☐ Never ☐Always ☐Sometimes Which?___________________

Crisps/snacks packets ☐ Never ☐ Always ☐ Sometimes Specify which______________

Candy ☐ Never ☐ Always ☐ Sometimes Which?___________________

12. Do you perform any physical activity outside of school hours?

☐ YES ☐ NO

13. How would you describe your physical activity?
☐ Sedentary ☐ Moderate ☐ Active
☐ Very active ☐ I don’t know

THANK YOU VERY MUCH
